# Sensitization of epithelial growth factor receptors by nicotine exposure to promote breast cancer cell growth

**DOI:** 10.1186/bcr3055

**Published:** 2011-11-15

**Authors:** Takashi Nishioka, Hyun-Seok Kim, Ling-Yu Luo, Yi Huang, Jinjin Guo, Chang Yan Chen

**Affiliations:** 1 Beth Israel Deaconess Medical Center, Harvard Medical School, 330 Brookline Avenue, Boston, MA, 00215, USA; 2 Yonsei University College of Medicine, 134 Shin-chon Dong, Seodaemoon-gu, Seoul, 120-749, Republic of Korea; 3 The First Hospital of Nanchang University, 17 Yongwai Zhengjie, Nanchang, 330047, China

## Abstract

**Introduction:**

Tobacco smoke is known to be the main cause of lung, head and neck tumors. Recently, evidence for an increasing breast cancer risk associated with tobacco smoke exposure has been emerging. We and other groups have shown that nicotine, as a non-conventional carcinogen, has the potential to facilitate cancer genesis and progression. However, the underlying mechanisms by which the smoke affects the breast, rather than the lung, remain unclear. Here, we examine possible downstream signaling pathways of the nicotinic acetylcholine receptor (nAChR) and their role in breast cancer promotion.

**Methods:**

Using human benign MCF10A and malignant MDA-MB-231 breast cells and specific inhibitors of possible downstream kinases, we identified nAChR effectors that were activated by treatment with nicotine. We further tested the effects of these effector pathways on the regulation of E2F1 activation, cell cycle progression and on Bcl-2 expression and long-term cell survival.

**Results:**

In this study, we demonstrated a novel signaling mechanism by which nicotine exposure activated Src to sensitize epidermal growth factor receptor (EGFR)-mediated pathways for breast cancer cell growth promotion. After the ligation of nAChR with nicotine, EGFR was shown to be activated and then internalized in both MCF10A and MDA-MB-231 breast cancer cells. Subsequently, Src, Akt and ERK1/2 were phosphorylated at different time points following nicotine treatment. We further demonstrated that through Src, the ligation of nicotine with nAChR stimulated the EGFR/ERK1/2 pathway for the activation of E2F1 and further cell progression. Our data also showed that Akt functioned directly downstream of Src and was responsible for the increase of Bcl-2 expression and long-term cell survival.

**Conclusions:**

Our study reveals the existence of a potential, regulatory network governed by the interaction of nicotine and nAChR that integrates the conventional, mitogenic Src and EGFR signals for breast cancer development.

## Introduction

Tobacco smoke is strongly linked to the onset of various types of human malignancies. According to epidemiological studies, about 30% of cancer deaths every year in the United States are associated with exposure to tobacco smoke or tobacco products, indicating the importance and urgency for cessation of active and passive cigarette smoke [1,2]. Tobacco smoke is known to be the main cause of lung, head and neck tumors [1,3-5]. Recently, evidence has been emerging for the increasing breast cancer risk associated with tobacco smoke exposure [6-9]. Nicotine, one of the important constituents of tobacco interacts with nicotine acetylcholine receptors (nAChR) and functions in either the motor endplate of muscle or at the central nervous system for the establishment of tobacco addiction [10-13]. Studies also showed that nAChR is expressed in various non-neuronal cells and the ligation of the receptor activates various intracellular signaling pathways in these cells, suggesting that nicotine has the potential to regulate cell proliferation [14-16]. It was reported that nicotine potently induced secretion of different types of calpain from lung cancer cells, which then promoted cleavage of various substrates in the extracellular matrix to facilitate metastasis and tumor progression [5]. In mammary epithelial or tumor cells, the exposure of nicotine initiated a signaling cascade that involved PKC (protein kinase C) and cdc42, and consequently accelerated cell migration [7]. Furthermore, the anti-apoptotic property of nicotine in breast cancer cells has been demonstrated to be through upregulation of Bcl-2 family members [8]. The addition of nicotine desensitized MCF7 cells to doxorubicin-mediated cyctoxicity [17]. All these data indicate that nicotine plays a positive role in the regulation of cell growth and survival. However, the underlying mechanisms of nicotine in facilitating mitogenic activities remain unclear.

nAChR consists of nine α-subunits (α2 to 10) and two β-subunits (β2 and 4) [10-13]. The subunits of nAChR form heteromeric or homoeric channels in different combinations in neuronal cells, which are highly Ca^++ ^permeable to allow the penetration of Ca^++ ^flux [10-13]. Upon the engagement with nAChR in non-neuronal cells, nicotine activates calmodulin-dependent protein kinase II, PKC, phosphodylinositol-3-kinase (PI3K)/Akt and Rac family that are often involved in the regulation of cell growth, adhesion or migration [7,18-20]. The activation of nicotine receptors was also shown to trigger Ras/Raf/MEK/ERK--Ras/Raf/MEK (mitogen-activated protein kinase)/ERK (extracellular-signal-reguated kinase)-- signaling [7,21,22]. In addition, the involvement of nicotine in the activation of the tyrosine kinase JAK-2 (Janus Kinase-2) and transcription factor STAT-3 (Signal Transducer and Activator of Transcription-3) in oral keratinocytes was also observed [22].

The epidermal growth factor receptor (EGFR) is a transmembrane protein receptor that possesses an intrinsic tyrosine kinase activity [23,24]. The EGFR family consists of several members, including EGFR, ERBB2/HER2/NEU, ERBB3 and ERBB4. The ligation of EFGR activates mitogenic-related signaling pathways, leading to various cellular responses. An increased level of mutation of EGFR has been detected in many human tumors, including breast cancer, which were often accompanied with a poor prognosis [25,26]. Upon growth factor stimulation, EGFR undergoes conformational changes and being phosphorylated, followed by being internalizated [24-26]. EGFR signaling subsequently mobilizes multiple signaling cascades, including MAPK (microtubule-associated protein kinase), PI3K (phosphodylinositol-3-kinase) and STAT (signal transducer and activator of transcription) pathways. However, a specific biological outcome, following EGFR activation, is determined by cross-talk or cooperation of its downstream effectors and parallel pathways.

As with EGFR, nAChR subunits appear to be activated through tyrosine phospohrylation [18,27]. Using *Xenopus *oocytes, neuroblastoma or other types of cells, it was shown that the α7 subunit of nAChRs was regulated by tyrosine phosphorylation and Src family kinases [18]. The treatment of colon cancer cells with nicotine activated c-Src as well as augmented EGFR expression [28]. Furthermore, in the colon cancer xenograft model, inhibitors of EGFR and Src dramatically blocked the tumor formation promoted by nicotine injection [29]. All studies suggest the existence of cooperation between nAChR and EGFR.

During the process of tumor initiation and progression, aberrant growth signaling plays an important role in the perturbation of growth restriction and cell cycle checkpoints. Numerous factors play a role in the regulation of this process, which includes growth factors, kinases, phosphatases as well as extracellular matrix components. Growth receptors, when interacting with corresponding ligands, initiate the process of cell cycle progression and migration in cells. In order to successfully transmit signaling from the membrane to the nucleus, receptors appear to communicate with each other to modulate the magnitude of signaling cascades and further activate transcription factors for the promotion of various biological processes. Nicotine has been demonstrated to induce nAChR phosphorylation, which further stimulated the dissociation of E2F1 from Rb and subsequent binding to cdc6 and cdc25A promoters for cell cycle progression in lung cancer cells [18]. These events which are induced by nicotine are most likely responsible for the increase of breast cancer risk by active or passive tobacco smoking.

In this study, we demonstrate a novel signaling mechanism whereby nAChR promotes breast cell growth through the sensitization of EGFR-mediated signaling. Upon nicotine-induced EGFR activation, Src, Akt and ERK1/2 were phosphorylated in MCF10 and MDA-MB-231 breast cancer cells, leading to the upregulation of E2F-1, Bcl-2 expression, and long-term cell survival. In this process, Src functioned directly downstream of nAChR to activate EGFR/ERK1/2 as well as Akt pathways, respectively. The identification of the cross-talk between nicotine and EGFR connected through Src provides a new insight into the potential carcinogenic effect of tobacco smoke on the breast.

## Materials and methods

### Cells, reagents and infection procedure

Human benign MCF10A and malignant MDA-MB-231 breast cancer cells were purchased from ATCC (Manassas, VA, USA). MCF10A cells were cultured in DMEM/F12 medium supplemented with 5% donor horse serum and antibiotics without growth factors. MDA-MB-231 cells were maintained in Dulbecco's Modified Eagle's Medium (DMEM) with 10% fetal calf serum, 4 mM L-glutamine and antibiotics. *dn-Src *or *dn-Akt *(dominant-negative *Src *or *Akt*) was inserted into MSCV (murine stem cell virus) retroviral vector and subsequently transiently infected into the cells.

Nicotine and the nAChR inhibitor mecamylamine hydrochloride (MCA) were purchased from Sigma-Aldrich, Inc. (St. Louis, MO, USA). The Akt inhibitor KP372-1 and the ERK inhibitor PD98059 were obtained from EMD Chemicals Inc. (San Diego, CA, USA). The antibodies were purchased from BD Parmingen (La Jolla, CA, USA).

The procedure for the infection with genes inserted in the MSCV retroviral vector was detailed in the User Manual provided by the company (BD Biosciences Clontech, Heidelberg, Germany). Briefly, after co-transfected expression vector, *Gag *and *Env *constructs, PT67 cells were grown for 48 hours. Subsequently, the medium was collected for the infection.

The experiments performed in this study do not require Institute Ethics Board approval, because only commercially available cell lines were used.

### Immunoblotting

Following treatment, cell lysates were prepared and proteins were separated by SDS-PAGE gels. Membranes were incubated with the designated primary antibody (1:1000 for all antibodies) overnight in a cold-room at 4°C. Bound primary antibodies were reacted with corresponding second antibodies for 2 hours and detected by chemiluminescence. The anti-phosphor-EGFR (tyr-1068), EGFR, phosphor-E2F, E2F, phosphor-Src, Src and Bcl-2 antibodies were purchased from Santa Cruz, Inc. The anti-phosphor-PDGFRβ, PDGFRβ, phosphor-ERK1/2, ERK1/2, phosphor-Akt and Akt antibodies were from Cell Signaling Technology, Inc, Donvers, MA, USA

### GST/Grb2 pull-down assay

GST/Grb2 fusion protein was purchased from Invitrogen. After treatments, cell lysates were incubated with the fusion protein (1 μg) immobilized on glutathione-sepharose beads as indicated in the protocol provided by the company. Bound proteins were washed and subjected to SDS-PAGE.

### ChIP assay

After treatments, cells were cross-linked with 1% formaldehyde for 15 minutes at room temperature. The cross-linking was stopped by the addition of glycine. Cells were harvested and sonicated. Lysates were immunoprecipitated with the corresponding antibodies. The different bindings of E2F1, Rb to cdc25A were analyzed by PCR. The sequences of the primers used are: cdc25A promoter size of 209 bp (5'-tctgctgggagttttcattgacctc and 3'-ttggcgccaaacggaatccaccaatc); c-Fos promoter size of 209 bp (5'-tgttggctgcagcccgcgagcagttc and 3'-ggcgcgtgtcctaatctcgtgagcat) [18]. PCR products were resolved on a gel.

### [^3^H]thymidine incorporation

Cells were grown in Petri dishes until 60% to 70% confluence and five wells were for the control and each treatment. The cells were cultured in medium containing 0.5% serum for 24 hours. Subsequently, the cells were grown in fresh medium containing 0.5% of serum plus 4 μCi/ml of [^3^H]thymidine (Perkin Elmer Life Sciences, Waltham, MA, USA) with or without various treatments. The cells were labeled for 8 hours at 37°C. After precipitation with cold 10% trichloroacetic acid, the cells were dissolved in 0.5 ml of 0.1 M NaOH overnight at 4°C. The amount of radioactivity in each sample was counted using a scintillation machine.

### Cell proliferation assay

Cells (2 × 10^5^) were plated in 12-well plates and cultured in medium containing 0.5% serum, which is designated as day 1. Subsequently, the cells with or without nicotine treatment were grown for another three days. The numbers of viable cells were determined by trypan blue staining and counted daily using a hemocytometer.

### Colony formation assay

Cells (250 cells/plate) were seeded in 100 mm-Petri dishes and cultured in growth medium containing nicotine alone or nicotine plus other inhibitors for ten days. The medium with nicotine or its combination with other inhibitors was changed every four days. After staining, the numbers of colony were counted.

### Statistical analysis

Three to five independent repeats were conducted in all experiments. Error bars represent these repeats. A Student's T test was used and a *P *value of < 0.05 was considered significant.

## Results

### EGFR was activated and internalized in breast cancer cells following treatment with nicotine

**Figure 1:**
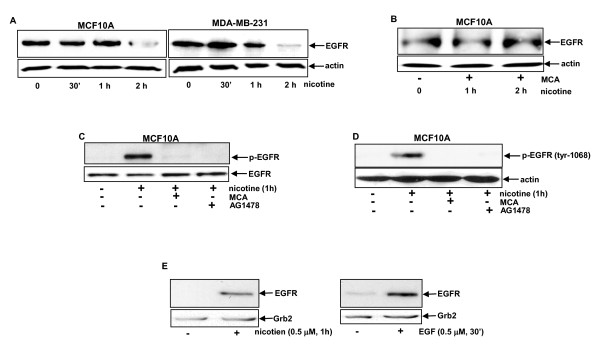
**Activation of EGFR in human breast cancer cells after nicotine treatment**. (**A**) Human MCF10A and MDA-MB-231 cells were treated with nicotine (0.5 μM) for various times and cell lysates (without nucleus) were extracted for testing EGFR expression by immunoblot. The blot was re-probed with an anti-β-actin Ab for loading control. (**B**) Expression of EFGR in nicotine-treated MCF10A cells in the presence of MCA (50 nM) was analyzed by immunoblot. The blot was re-probed with an anti-β-actin antibody for loading control. (**C **and **D**) MCF10A cells were treated with MCA (50 nM) or AG1478 (100 nM) for 15 minutes prior to 1 hour of nicotine exposure. The phospohrylation pattern of EGFR in nicotine-treated MCF10A cells was examined by immunoprecipitation with anti-EGFR antibody and immunoblotting with the anti-phosphor-tyrosine antibody, or immunoblotting with the anti-phosphor (Tyr 1068) antibody. The blot was re-probed with an anti-EGFR antibody for loading control.(**E**) With or without treatment with nicotine (left panels) or EGF (right panels), the cell lysates were extracted and precipitated with glutathione S-transferase/growth factor receptor-bound protein 2 (GST/Grb2) fusion protein. Subsequently, the precipitates were subjected to immunoblotting. The blot was also re-probed with anti-Grb2 antibody for the control.

Upregulation of EGFR signaling plays an important role in breast cancer development and cooperation between nAChR and EGFR has been suggested in cancer progression [29,30]. However, the mechanisms by which cigarette smoke or nicotine exposure promotes breast tumorigenesis remain unclear. This study aimed at investigating the existence of a cross-talk between nAChR and EGFR for the promotion of breast cancer growth. After treatment with nicotine at different time points, a cell lysate (without the nucleus) was prepared from human breast cancer MCF10A or MDA-MB-231 cells and the expression of EGFR was then tested by immunoblotting (Figure 1A). The levels of EGFR in the lysate from cells treated with nicotine for 30 minutes or 1 hour were similar to those in untreated cells. Interestingly, EGFR (> 85%) became undetectable in the lysate extracted from MCF10A cells treated with nicotine for 2 hours. In the presence of MCA (a nAChR inhibitor), the level of EGFR in the same cells subjected to the same treatment did not decline (Figure 1B). It appears that the disappearance of EGFR was specifically triggered by nicotine treatment.

Upon motigenic activation, EGFR is often seen to be phosphorylated at its tyrosine residues and then being terminated [23]. Since EGFR in the cells became undetectable 2 hours after nicotine exposure, the phosphorylation status of the receptor at an earlier time point (1 hour) in the treatment was examined (Figure 1C). The lysates from untreated or treated cells were immunoprecipitated with an anti-EGFR antibody and then subjected to immunoblotting, using the anti-phosphor-tyrosine antibody (Figure 1C). The phosphorylated EGFR in MCF10A cells was recognized by the antibody 1 hour after the treatment, which was abrogated by the addition of either MCA or AG1478 (an EGFR inhibitor). For confirmation purposes, the phosphor-(Tyr1068)-EGFR antibody was also used to detect EGFR phosphorylation status and a similar result as that shown in Figure 1C was obtained (Figure 1D). It is known that through association with Grb2, active EGFR triggers a cascade of its downstream effectors [31]. To test whether nicotine-activated EGFR was able to bind to Grb2, MCF10A cells were treated with nicotine or EGFR and immunoprecipitation was then performed (Figure 1E). The receptor was found to be bound to a GST (glutathione-S-transferase)/Grb2 fusion protein in either nicotine- or EGF-treated cells, but not in untreated control cells. The data further suggested that the ligation of nicotine with nAChR stimulated EGFR.

### EGFR in breast cancer cells is specifically activated by nicotine ligation

**Figure 2:**
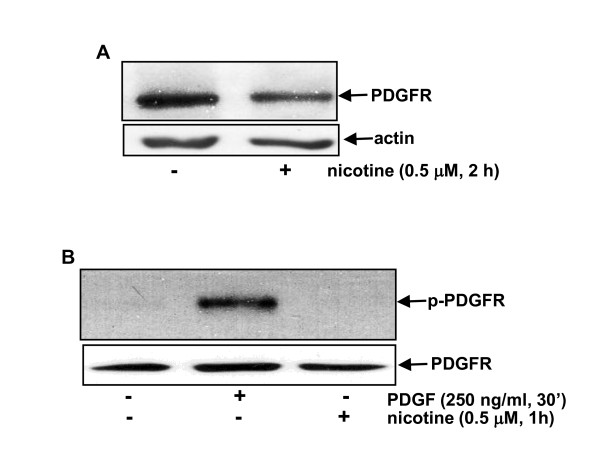
**Activation status of PDGFR in MCF10A cells in response to nicotine treatment**. (**A**) After the treatment with nicotine, the expression of PDGFR in MCF10A cells was analyzed by immunoblotting. The blot was re-probed with an anti-β-actin antibody for loading control. (**B**) Following the treatments, the expression of the phosphor-PDGFR in MCF10A cells was assayed and the blot was re-probed with an anti-β-actin antibody for loading control.

To test if nAChR activation might globally sensitize cell surface receptors, MCF10A cells were treated with nicotine for 2 hours and immunoblotting was performed using anti-platelet growth factor β subunit (PDGFRβ) antibody (Figure 2A). Unlike EGFR, the level of PDGFR in nicotine-treated cells was unchanged. To further test the activation status of PDGFR, MCF10A cells were treated with PDGF for 30 minutes or nicotine for 1 hour and immunoblotting was performed using the anti-phosphor-PDGFRβ antibody (Figure 2B). The receptor was phosphorylated after treatment with PDGF, as expected. However, the phosphor-PDGFRβ was unable to be visualized by the antibody in nicotine-treated cells. These data suggested that the sensitization or internalization of EGFR in breast cancer cells is specifically induced by nicotine exposure.

### Downstream effector kinases were activated after nicotine treatment

**Figure 3:**
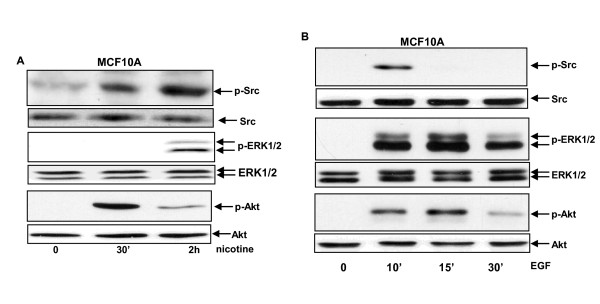
**Activation status of Src, ERK1/2 and Akt in MCF10A cells following nicotine exposure**. (**A**) After nicotine treatment for different time periods, the phospohrylation form of Src, ERK1/2 or Akt in MCF10A cells was analyzed by the corresponding antibodies, using immunoblot. (**B**) The expression of the phosphorylated Src, ERK1/2 or Akt in EGF-treated cells.

It is known that tyrosine kinase Src is not only downstream of EGFR but also of nAChR [29,30]. Thus, the activation status of Src in MCF10A cells was examined after nicotine treatment at different time points (Figure 3A, upper two lanes). Src was not activated in untreated cells. However, this kinase was phosphorylated 1 hour after nicotine exposure and an increased amount of the active form of this kinase was present in the cells 2 hours following treatment.

Akt and ERK1/2 often exert as receptor downstream effectors of EGFR or Scr in mitogen-induced responses. The phosphorylation status of these kinases was also examined after MCF10A cells were treated with nicotine. Two hours after nicotine treatment, the phosphorylated forms of ERK1 and 2 were detected by the antibody in the cells (Figure 3A, middle two lanes). Also, a high level of phospohrylated Akt was detected by the antibody 1 hour after nicotine exposure and a smaller amount of the phosphorylated protein was seen at 2 hours of the treatment (Figure 3A, bottom two lanes). The same activation patterns of these kinases were seen in nicotine-treated MDA-MB-231 cells (data not shown). In comparison, a fast activation pattern of these kinases was seen in response to EGFR treatment in the cells. Following the treatment with EGF for 10 or 15 minutes, Src, ERK1/2 or Akt was phosphorylated (Figure 3B). One hour after the treatment, these kinases were no longer active (data not shown). Since these kinases activated with different activation kinetics upon nicotine treatment, the results indicated that distinct mechanisms are involved in the regulation of these nAChR downstream effectors.

### nAChR, via Src, activates EGFR-dependent or -independent downstream pathways following nicotine treatment

**Figure 4:**
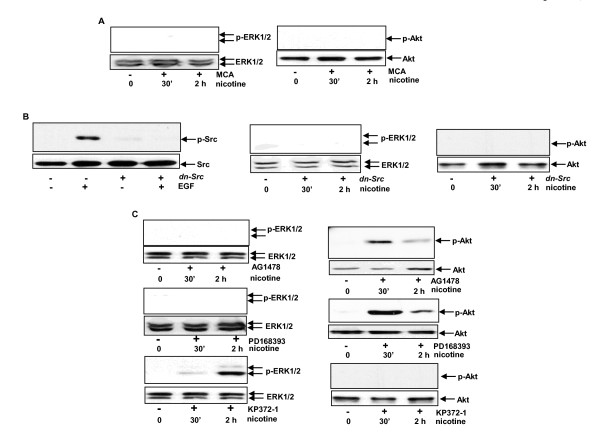
**The hierarchy order of nAChR downstream effectors in MCF10A cells**. **(A**) After the suppression of nAChR by MCA, the phosphorylation status of ERK1/2 or Akt was tested by immunoblot. (**B**) After transient transfection of *dn-src*, MCF10A cells were treated with EGF (100 ng/ml) for 15 minutes and the expression of the phosphorylated form of Src was then examined by immunoblot (left panels). The expression of phosphorylated ERK1/2 or Akt in nicotine-treated MCF10A cells with or without transiently expressing *dn-src *was analyzed by immunoblot (middle and right panels). (**C**) The cells were treated with AG1478 (100 nM), PD168393 (5 μM) or KP372-1 (1 μM) for 15 minutes, prior to nicotine exposure. Subsequently, the expression of the phosphorylated ERK1/2 or Akt was analyzed. -the full names of all abbreviations (including the inhbitors) are provided in the text.

Since c-Src, Akt, and ERK1/2 in the cells were activated after nicotine treatment, it was possible that these kinases were subjected to different regulations. To test this, we treated MCF10A cells with MCA (a nAChR inhibitor), and then with nicotine for various time points (Figure 4A). Neither ERK1/2 nor Akt was phosphorylated in nicotine-treated cells after the blockade of nAChR. A dominant-negative *src *was then used to suppress Src. To verify if the *dn-src *had an inhibitory effect on endogenous Src, we transiently transfected the construct into MACF10A cells and treated the cells with EGF (a known Src activator). Indeed, the introduction of *dn-src *efficiently blocked EGF-induced Src phosphorylation (more than 90%) (Figure 4B, left panels). After dn-src was transiently transfected into the cells, the phosphorylated form of ERK1/2 or Akt could not be detected in nicotine-treated cells (Figure 4B, middle and right panels). We then treated MCF10A cells with AG1478 prior to nicotine exposure. The inhibition of EGFR by the inhibitor prevented nicotine-mediated phosphorylation of ERK1/2 (Figure 4C, upper panels), but had no effect on nicotine-induced Akt activation. Subsequently, the cells were exposed to PD168393 (an ERK inhibitor) (Figure 4C, middle panels) or KP372-1 (an Akt inhibitor) (Figure 4C, bottom panels), prior to the addition of nicotine. The inhibitors suppressed the activation of the corresponding kinases, respectively. The data suggested that Src is downstream of nAChR and responsible for the sensitization of EGFR or Akt pathway. However, ERK1/2 signaling appeared to be controlled by EGFR in nicotine-mediated, growth-related action.

### E2F1 activity was upregulated by nicotine through EGFR pathway

**Figure 5:**
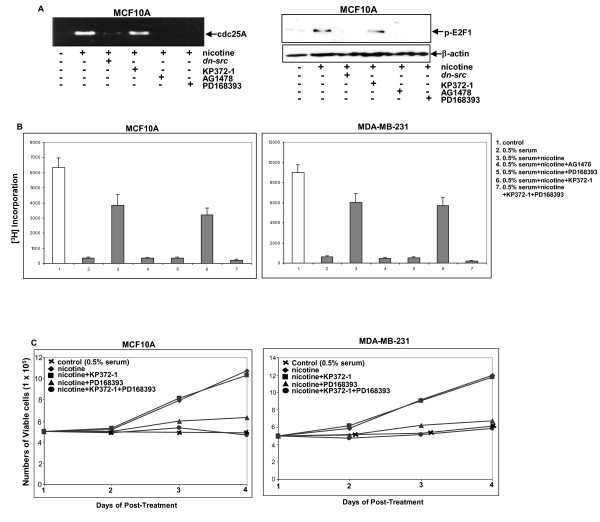
**Involvement of E2F1 in cell growth promotion by nicotine**. (**A**) Left panel: ChIP analysis of nicotine-treated MCF10A cells in the presence or absence of *dn-src*, PD168393, AG1478 or KP372-1. Sonicated, cross-linked chromatin was immunoprecipitated with an anti-E2F1 antibody, and the purified DNA was assayed by PCR with the primers for *cdc25A *promoter. Right panels: After the same treatments as described above, cell lysates were prepared and subjected to immunoblotting with the anti-phosphor-E2F antibody. (**B**) After different treatments, [^3^H]thymidine incorporation in the cells was measured. (**C**) After being serum-starved for 24 hours, the cells were cultured in the medium containing 0.5% serum plus various inhibitors, and the numbers of viable cells were counted for 4 days. Error bars represent SD (standard deviation), *n *= 5.

EGF/EGF-related signals are able to activate downstream pathways to inactivate Rb, leading to the release of E2F from its sequestration and the entry of cells to S phase of the cell cycle [32]. To test whether nicotine treatment could affect E2F1 activity in breast cancer cells, a ChIP assay was conducted to analyze the occupancy of E2F1 on its responsive *cdc25A *promoter (Figure 5A, left panel). Stimulation with nicotine for 2 hours induced the association of E2F1 with *cdc25A *promoter in MCF10A cells. The blockade of Src by *dn-src *or suppression of EGFR signaling by AG1478 abolished the binding of E2F1 to the promoter induced by nicotine. Consistently, the inhibition of Akt by KP372-1 did not affect E2F1 association with the promoter in nicotine-treated cells and the addition of PD168393 completely interfered with the binding. The promoter of *c*-*Fos *was used as the control in the ChIP assay and E2F1 did not bind to this promoter in response to nicotine treatment (data not shown). The activation of E2F was also tested by immunoblotting using the anti-phosphor-E2F antibody and results similar to those found in the ChIP assay were obtained (Figure 5A, right panels). The results supported the notion that E2F1 activity induced by nicotine treatment was governed by nAChR/Src/EGFR/ERK1/2 signaling and Akt appeared to play no role in this nicotine-mediated, growth promotion.

Since E2F1 was activated by the EGFR/ERK1/2 pathway in our experimental setting, the [^3^H]thymidine incorporation assay was used to determine the role of this pathway in DNA uptake in nicotine-treated MCF10A and MDA-MB-231 cells (Figure 5B). After serum starvation for 48 hours, the cells were treated with nicotine or co-treated with various inhibitors in the presence of [^3^H]thymidine. Rates of DNA synthesis were then measured. Under serum depletion conditions, little [^3^H]thymidine incorporation was observed in the cells. A moderate amount of [^3^H]thymidine (about 5 to 6 fold) was incorporated in nicotine-treated cells under serum-starvation conditions. However, the addition of AG1478 or PD168393 blocked the nicotine-induced [^3^H]thymidine incorporation into the cell genomes. In comparison, KP372-1 treatment had a minimal, negative role in DNA synthesis promoted by nicotine. As expected, co-treatment of PD168393 and KP372-1 completely suppressed the incorporation of [^3^H]thymidine.

Next, the effect of Src or Akt on cell growth in response to nicotine exposure was assayed by cell proliferation analysis. After 24 hours of serum starvation, MCF10A or MDA-MB231 cells in the medium containing 0.5% serum were treated with PD168393, KP372-1 or infected with *dn-src*, prior to nicotine exposure, and the number of cells was then counted for four consecutive days (Figure 5C). MCF10A or MDA-MB231 cells did not grow under serum-depletion conditions. However, the numbers of the cells were increased at day 2 after the treatment. The addition of PD168393 significantly prevented nicotine-mediated growth promotion. In comparison, KP372-1 had no negative effect on nicotine-mediated growth promotion. Again, concurrent treatment with KP372-1 and PD168393 completely blocked the nicotine-mediated effect on the growth of MCF10A and MDA-MB-231 cells. The data further supported the notion that nicotine may sensitize EGFR/ERK1/2/E2F1 signaling to promote cell growth.

### Akt was involved in the regulation of cell survival upon nicotine treatment

**Figure 6:**
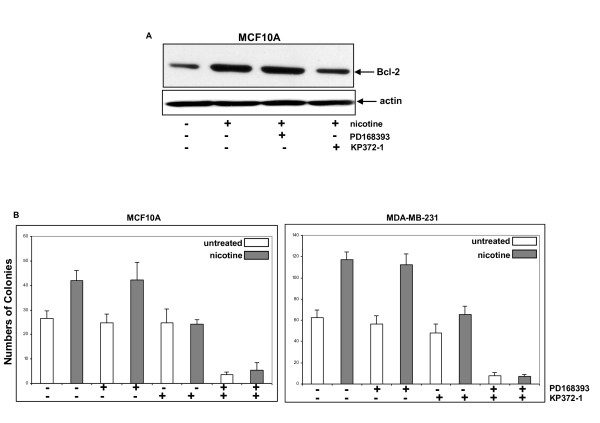
**Long term cell survival promoted by nicotine exposure**. (**A**) MCF10A cells were co-treated with different inhibitors and nicotine for 2 days. Subsequently, the expression of Bcl-2 was assayed by immunoblot. The blot was re-probed with an anti-β-actin antibody for loading control. (**B**) The cells (250 cells/per dish) were seeded and grown in the medium containing nicotine plus different inhibitors. The number of colonies was counted 12 days later. Error bars represent SD (standard deviation), *n *= 5.

Persistent nicotine exposure was shown to upregulate Bcl-2, which enhances cell survival as well as resistance of cancer cells to chemo-drugs [33-36]. To test how nicotine-mediated effector pathways were involved in the regulation of Bcl-2 or cell survival, MCF10 cells were co-treated with various inhibitors and nicotine for two days and the expression of Bcl-2 was assayed by immunoblotting (Figure 6A). The level of Bcl-2 expression in the cells was increased (about 2 fold) after nicotine treatment, which was not affected by its co-treatment with PD168393. Interestingly, this nicotine-mediated upregulation of Bcl-2 expression in the cells was blocked by co-treatment with KP372-1. A similar result was obtained in MDA-MB231 cells (data not shown).

To determine the effect of various nicotine-mediated signaling pathways on long-term cell survival, a colony formation assay was performed (Figure 6B). After being seeded, MCF10A and MDA-MB-231 cells formed colonies 12 days later, and the addition of nicotine stimulated the ability of the cells to form colonies (about 2 fold). Treatment with PD168393 or KP372-1 alone had no obvious effect on the formation of colonies of the cells. The co-treatment of nicotine with KP372-1, but not with PD168393 significantly reduced the numbers of the cells that formed colonies. Concurrent treatment with PK372-1 and PD168393 completely blocked MCF10A or MDA-MB-231 cells from generating colonies, with or without nicotine exposure. Overall, the data indicated that Akt might be responsible for nicotine-promoted cell survival.

## Discussion

Cigarette smoke contains a variety of genotoxic carcinogens, many of which are derivatives of nicotine that are formed during the curing of tobacco [1-3]. The direct link between cigarette smoke and the onset of lung cancer has long been established. Although the correlation of the smoke with other types of cancer, in particular breast cancer, has been suggested by epidemiological investigations, the underlying molecular mechanisms by which cigarette smoke promotes breast cancer genesis and progression remain unclear. It is known that nAChR is widely expressed in neurons and neuromuscular junctions, but is also present in various non-neuronal organs, tissues or cells, such as epithelial cells from different organs and endothelial cells. Ligation of nAChR has been shown to facilitate cell growth and promote pro-survival activities in lung cancer or other types of malignant cells [4-6]. We previously demonstrated that exposure to nicotine augmented the migration or invasion ability of benign or malignant breast cancer cell lines, in which PKC and cdc42 played a crucial role [7]. As the continuation of the investigation of the role of nicotine exposure in breast tumorigenesis, we found that the engagement of nicotine with nAChR sensitized EGFR signaling via Src, resulting in the activation of ERK1/2 and upregulation of E2F1 transcriptional activity. We also found that the inhibition of nAChR or Src abrogated the promotion of cell proliferation conferred by nicotine treatment. Furthermore, in response to nicotine treatment, ERK1 and 2 functioned downstream of EGFR and the suppression of these kinases prevented the nicotine-mediated activation of E2F1 and DNA synthesis. We also showed that Akt appeared to be directly activated by Src in nicotine-governed action and responsible for upregulated Bcl-2 expression and increase cell survival activity. Collectively, these findings identified the novel intracellular targets Src/Akt and EGFR/ERK1/2 that are differentially affected by nicotine exposure to facilitate breast cancer progression.

Since there is a lack of understanding about the underlying molecular mechanisms by which tobacco smoke promotes turmorigenesis in other organs of human body, rather than in the lung, nicotine has become a major object of investigation, because it exists in high concentrations in the blood stream of first-, heavy second-hand smokers and nicotine users [37-39]. Although nicotine is not a conventional carcinogen, this tobacco smoke-related compound has been shown to induce the secretion of growth factors (such as bFGF, TGF-α, calpains, and VEGF), resulting in the activation of Raf, Akt or PKC pathways for the growth promotion of lung epithelial or cancer cells and upregulation of Bcl-2 signaling that is responsible for the increase in the resistance to anti-cancer therapies [35,36]. The binding of nicotine to nAChR initiated the activation of Src tyrosine kinase that further mediated cell cycle progression of non-small cell lung cancer (NSCLC) [18]. Our current study demonstrated that exposure of human breast benign or malignant cancer cells to nicotine induced the phosphorylation of Src that augmented cell growth- and survival-related signaling. As a substance, nicotine is able to diffuse rapidly into various organs and tissues. Thus, it is conceivable that this major component of tobacco smoke in the blood stream can efficiently reach the breast and bind to nAChR on the surface of breast epithelial or cancer cells, which provides a growth advantage locally. Indeed, studies have demonstrated that cancer patients who were smokers or nicotine users were more resistant to chemotherapy and had increased metastasis of breast cancer. Furthermore, nicotine was also reported to augment the proliferation of cell lines derived from gastric, colon, bladder or pancreatic tumors [14-16]. Therefore, the interaction of nicotine and nAChR is an un-neglected factor in the regulation of the growth in different tissues or organs.

EGFR belongs to a family of the receptor tyrosine kinases and functions as a mediator to transmit cell signaling initiated by extracellular growth factors to the nucleus. Overexpression of EGFR or other family members is frequently found in human tumors of epithelial origin. Targeting EGFR family members has been attractive for developing new therapeutics with promising clinical results [23-26]. In our current investigation, we demonstrated that EGFR was activated and subsequently internalized in breast cancer cells in response to nicotine treatment, accompanied by the cascade of the phosphorylation of several intracellular effector kinases. Among these kinases, Src acted as a key regulator to link nAChR signaling to EGFR and ERK1/2. In nicotine-treated neuroblastoma or *Xenopus *oocytes cells, the α7 subunit of nAChR has been shown to undergo tyrosine phosphorylation and Src was responsible for the activation of this subunit of the receptor [18]. Using *in vitro *and xenograft assays, it was also reported that the levels of Src and EGFR in colon cancer cells were significantly increased following nicotine exposure [18]. Our experiments showed that Src functions as a key downstream effector of nAChR and links nicotine signals to EGFR and ERK1/2 to promote transient cell growth activities.

By studying the mechanisms of nicotine-mediated cell growth promotion, we revealed that a cross-talk occurred specifically between two important cell surface receptors: nAChR and EGFR. This is the first demonstration of nicotine-induced sensitization of EGFR in benign and malignant breast cancer cells. Intriguingly, we found that in nicotine-mediated action, EGFR activation led to an increase of E2F1 activity, resulting in the promotion of DNA synthesis and cell proliferation. In this process, EGFR appears as a rate limiting factor and ERK1/2 functions as an executor of the cell growth program. Previously, we established that exposure to nicotine activates Raf and PKC pathways in Rat or murine lung epithelial or cancer cells, which facilitate the genesis and development of tumors [23-26]. EGFR has been shown to mediate at least two pathways in cancer cells: the cytosolic and the nuclear pathways. Emerging evidence indicates that upon activation, some of EGFR or its family members in cancer cells relocate to the nucleus, where they participate in the regulation of gene transcription, cell cycle checkpoints and DNA repair. It is still under investigation whether EGFR upon nicotine treatment in our experimental setting translocates to the nucleus or is degraded. The present data suggest that upon nicotine exposure, EGFR appears to play a significant role in breast tumorigenesis.

Tobacco smoke or nicotine can reduce the efficacy of chemo-treatments and increase cancer onset, development or recurrence. Studies showed that in response to nicotine exposure, cancer cells became resistant to cytotoxicity triggered by anti-cancer drugs. Bcl-2 was reported to play an important role in nicotine-induced anti-apoptotic or pro-survival activities [35,36]. It was demonstrated that nicotine treatment significantly protected breast cancer cells against the cytotoxicity of doxorubicin [35,36]. Here, we determined that Bcl-2 is one of the targets of nicotine exposure. Our study also demonstrated that Akt was involved in the regulation of Bcl-2 expression and responsible for the long-term survival of the breast cancer cells. Together, it seems that nicotine, through activation of Src and Akt, promotes anti-apoptotic or pro-survival activities in breast cancer cells. Thus, Src and Akt pathways might be the intracellular targets for improving the treatment efficacy of breast cancer patients who are active or passive smokers or nicotine users.

## Conclusions

In summary, our findings suggest that Src and EGFR play pivotal roles in regulating nicotine-treated breast cancer cell proliferation and survival. The molecular mechanisms of the activation of Src and EGFR in nicotine-mediated action involve ERK1/2/E2F1 and Akt/Bcl-2 pathways. The cooperation of these pathways causes a full magnitude of the promotion of cell growth and survival, which are attractive targets for developing better treatments for breast cancer.

## Abbreviations

nAChR: nicotinic acetylcholine receptor; EGFR: epidermal growth factor receptor; Src: sarcoma; ERK1/2: extracellular signal regulated kinases 1 and 2; PI3K: phosphodylinositol-3-kinase; JAK-2: Janus kinase 2; STAT-3: signal transducers and activators of transcription protein-3; Rb: retinoblastoma protein; NSCLC: non-small cell lung cancer; Raf, PKC: protein kinase C; MEK: mitogen activated protein kinase; MAPK: microtule-associated protein kinase; PI3K: phosphodylinositol-3-kinase; PDGFRβ: platelet growth factor β subunit.

## Competing interests

The authors declare that they have no competing interests.

## Authors' contributions

TK, HK, LL, YH and JG carried out the experiments and initial analysis and interpretation of the data. CC conceived and designed the studies, made further data interpretations and wrote the manuscript. All authors approved the final version of the manuscript.
